# Current Understanding of the Role of the Brd4 Protein in the Papillomavirus Lifecycle

**DOI:** 10.3390/v5061374

**Published:** 2013-05-30

**Authors:** Alison A. McBride, Moon Kyoo Jang

**Affiliations:** Laboratory of Viral Diseases, National Institute of Allergy and Infectious Diseases, National Institutes of Health, Bethesda, MD 20892, USA; E-Mail: jangmk@niaid.nih.gov

**Keywords:** Brd4, BET protein, HPV, papillomavirus, transcription, chromatin, replication, tethering, partitioning, bromodomain

## Abstract

The Brd4 protein is an epigenetic reader that is central to regulation of cellular transcription and mitotic bookmarking. The transcription and replication proteins of many viruses interact with Brd4. We describe the multiple roles of Brd4 in the papillomavirus lifecycle.

## 1. Introduction

**Figure 1:**
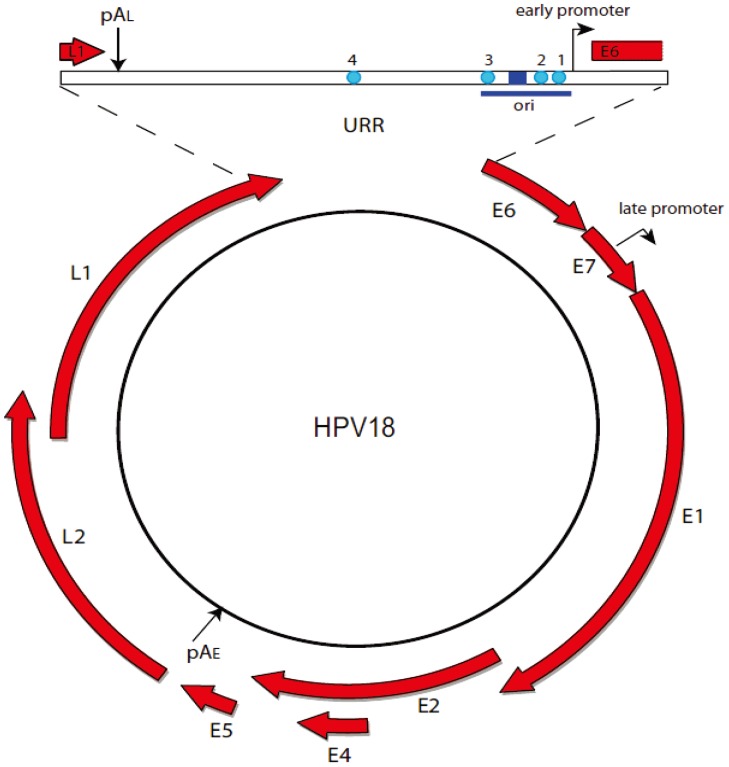
HPV18 genome. The circular dsDNA genome of HPV18 (7,857 bp) is shown. Viral open reading frames are depicted as red arrows. The URR (upstream regulatory region) is expanded to show transcription and replication regulatory elements. Binding sites for the E2 protein (cyan circles) and the E1 binding site (blue rectangle) are shown. The early promoter and replication origin (ori) are indicated.

Papillomaviruses (PVs) are an ancient group of viruses that have coevolved along with their hosts for millions of years. Each viral type infects only a particular host species and is trophic for a specific anatomical niche in the stratified epithelium of the skin or mucosa of the host. Papillomavirus infection is persistent and results in clinical outcomes, such as asymptomatic infection, verrucae, plantar and filiform warts and condylomata acuminata. A subset of oncogenic HPVs is associated with carcinomas of the oropharyngeal and anogenital tracts [1,2,3]. Despite the diversity of pathogenesis associated with papillomavirus infection, all papillomaviruses have similar small dsDNA genomes of approximately 8 kbp (see Figure 1), and each encodes only six to eight genes. Papillomaviruses rely on hijacking and manipulating host factors to maintain their lifestyle. 

One key, cellular regulatory protein hijacked by all papillomaviruses (and other viral families) is a cellular chromatin binding protein, Brd4. Brd4 is an essential cellular protein that binds and marks chromatin to regulate several transcriptional processes. In this review, we will summarize the current knowledge pertaining to the role of Brd4 in papillomavirus infections. 

## 2. Papillomaviruses

### 2.1. The Papillomavirus Lifecycle

The stratified epithelium of the skin or mucosa consists of several layers of keratinocytes in various stages of differentiation, overlying the basal layer of proliferative cells. Papillomaviruses gain access to cells in the basal layer through a microabrasion. They infect these keratinocytes, induce their proliferation by expression of the viral E7 protein and establish a persistent infection therein. The viral genome is maintained as a low copy, extrachromosomal element in the nucleus of these cells, and only low levels of viral gene products are produced. When the infected cells divide, some of the daughter cells begin the process of stratification and differentiation and progress towards the surface of the epithelium. The papillomavirus lifecycle is finely tuned to this process of differentiation; viral transcription and replication switches from early to late modes as the cell differentiates. Vegetative viral DNA replication is initiated in the mid-layers of differentiated cells, and capsid synthesis is confined to the most differentiated layers of cells.

### 2.2. Viral Transcription

Papillomavirus genomes can be divided into three regions (see Figure 1). The upstream regulatory region (URR) contains transcriptional enhancers and promoters and the origin of replication. The early region encodes the E (early) proteins that are expressed at early and intermediate stages of infection. Last is the late region that contains the L (late) genes. In the most undifferentiated cells, viral RNAs are transcribed from the early promoter, terminate at the early polyadenylation site (pAE) and encode the E6, E7, E1, E2, E4 and E5 proteins (reviewed in [4]). As the cells progress through differentiation, the late promoter is activated, resulting in an intermediate class of transcripts that use the late promoter and the early polyadenylation signal. These transcripts encode high levels of the E4, E1 and E2, the latter two being required for high level vegetative viral DNA replication in the mid‑layers of the epithelium. Exclusively late transcripts are expressed at an even later stage of differentiation; they use both the late promoter and late polyadenylation signal (pAL) and encode the L1 and L2 capsid proteins. Complex patterns of viral mRNA species are generated by the use of alternative splicing and viral gene expression is highly regulated by differential splicing and polyadenylation (reviewed in [4]). 

**Figure 2:**
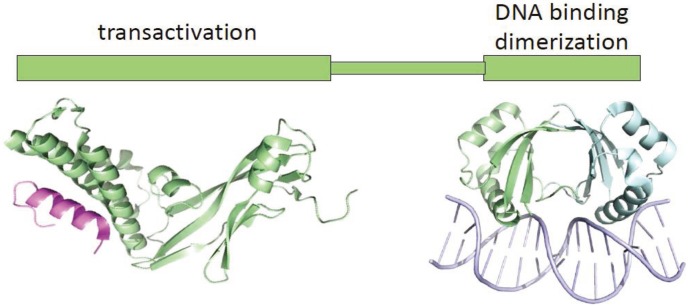
Structure of the papillomavirus E2 protein. The two domains of the E2 protein are shown. The transactivation domain structure is of HPV16 E2 bound to the Brd4 C-terminal motif (CTM). The Brd4 C-terminal peptide (residues 1343–1362) is shown in magenta. This structure is from the pdb file, 2NNU. The DNA binding domain is of the HPV18 DNA binding domain bound to DNA (shown in light purple) from the pdb file 1JJ4.

The E2 protein is the primary regulator of early viral transcription [5]. E2 consists of a conserved amino terminal “transactivation” domain linked to a C-terminal DNA binding/dimerization domain by a poorly conserved linker (see Figure 2) [6]. E2 specifically binds to a consensus sequence of ACCGNNNNCGGT, several copies of which are located in the viral URR [7] (see Figure 1). E2 can activate transcription from certain viral promoters [5,8,9,10], but the early viral promoter in the well-studied, oncogenic alpha-PVs is primarily repressed by E2 [11,12,13]. Early studies showed that E2 could repress the early promoter by sterically hindering the association of TBP and Sp1 with promoter elements adjacent to E2 binding sites 1 and 2 [14,15,16]. Subsequent studies have shown that much of this E2-mediated repression is mediated through interaction with factors that modulate cellular chromatin processes [17,18,19,20]. Disruption of E2-mediated repression (often due to HPV integration) results in increased expression of the E6 and E7 oncogenes [11,12,21]. In fact, reintroduction of the E2 protein into cervical cancer-derived lines (such as HeLa) leads to rapid senescence, as viability of these cells is completely dependent on E6 and E7 expression [22,23,24].

Shorter forms of the E2 protein that are lacking the transactivation domain also repress HPV transcription, in part by competing for binding to E2 binding motifs [25,26]. One of the best characterized is the E8^E2 protein (encoded from a spliced transcript), which also represses through the recruitment of chromatin-associated repressive factors [27]. As described in detail below, E2 interacts with the Brd4 protein, and this association is pivotal to transcriptional regulation of HPVs.

### 2.3. Disruption of E2 Function by HPV Integration

In the majority of HPV associated cancers, the HPV genome is integrated into the host genome (reviewed in [28]). The viral genome is most often integrated in such a way as to disrupt the E2 gene and, thus, alleviate transcriptional repression of the early viral promoter [22,23,24,29]. In turn, this causes dysregulation of the E6 and E7 oncogenes and promotes malignant progression [30]. 

### 2.4. Viral Replication

Papillomavirus replication requires the E1 protein, the E2 protein and the origin of replication [31,32,33]. E1 is the primary replication protein, an ATP-dependent helicase that binds specifically to the origin and unwinds it in a bidirectional manner to permit access by the cellular replication machinery (reviewed in [34]). E2 functions to load the E1 helicase onto the origin [35]. As depicted in Figure 1, the minimal origin consists of the E1 binding site flanked by E2 binding sites and overlaps elements regulating the early promoter. 

There are three modes of viral replication in the viral lifecycle. The first is a limited amplification that occurs when the virus particle infects a basal keratinocyte. The virus must undergo a few rounds of unlicensed DNA replication to establish the genome as a low copy, nuclear, extrachromosomal element. This phase requires E1, E2 and the minimal replication origin. The second phase of replication maintains the viral genome at a low copy number during division of the proliferating basal cells. This also requires E1, E2 and the minimal origin of replication, but there is an additional requirement for additional E2 binding sites in *cis* to the origin, thus implicating the E2 protein in maintenance replication [36]. Subsequent studies showed that the role of E2 was to tether the viral genomes to the host chromatin [37,38,39]. The interaction of E2 with Brd4 is crucial for the tethering function of many papillomaviruses and will be discussed in detail below. Further investigation showed that the E1 protein was not always necessary for maintenance replication and, presumably, in this situation viral replication, is initiated by cellular factors, while the genome is retained by the E2 protein [40,41]. 

The third phase of replication is vegetative amplification, when progeny virions are produced in large numbers in differentiated cells. This requires the E1 and E2 proteins, and their expression is upregulated in differentiated cells [42,43]. There is evidence that the mode of replication changes in differentiated cells [44], and incorporation of Rad51 into replication foci indicates that the virus may replicate using a recombination directed replication mechanism [45,46]. Furthermore, the cellular ataxia telangiectasia mutated (ATM) DNA damage response pathway is required for vegetative replication in differentiated cells [47]. Nuclear foci formed by either expression of the E1 and E2 proteins [48,49,50,51] or the replicating viral genome [46,47] recruit multiple cellular proteins required for the cellular DNA damage response and repair pathways.

### 2.5. Differences in Transcription and Replication among Papillomaviruses

To date, there are over 240 named papillomavirus genomes that have been classified into 37 different genera [52]. The best studied are human viruses from the alpha and beta genera that infect primarily the mucosa and skin, respectively. The human mu virus, HPV1, and the ungulate delta virus, BPV1, are also well characterized. While each of these viruses has a similar organization of genes to that of HPV18 (an alpha-PV) shown in Figure 1, the number and position of the E2 binding sites can vary considerably. BPV1 has 11 E2 binding sites in the URR and six elsewhere in the genome [53], while most human alpha viruses have only the four E2 sites shown in Figure 1. In BPV1 E2 is primarily an activator of transcription [5], while E2 predominantly represses the major early promoter of alpha-PVs.

All viruses require the minimal replication origin and the E1 and E2 proteins to initiate replication, but the requirements for maintenance replication are more complex. E2 binding sites are essential for initiation of replication and for transcriptional regulation making it very difficult to separate and elucidate the role of individual sites in maintenance replication of the viral genome. Maintenance replication is best understood for BPV1, where it has been shown that at least eight E2 binding sites are required for persistent replication [36]. In the alpha-PV HPV31, only three of the four E2 binding sites are required for maintenance replication of the viral genome [54]. In agreement with this finding, using a novel complementation assay, we find that a region encompassing the 3’ half of the URR of HPV18 (containing E2 binding sites 1–3) is sufficient for long-term maintenance in the presence of the E1 and E2 proteins [55]. 

## 3. The Brd4 Protein

### 3.1. Brd4 Structure and Function

Brd4 was first described as an unusual chromatin binding factor that remained bound to chromosomes throughout mitosis [56]. It is a member the BET (bromodomain and extra-terminal domain) family of chromatin binding proteins and, therefore, its name was changed from MCAP (mitotic chromosome-associated protein) to bromodomain containing protein 4 (Brd4) [57]. Brd4 is an essential protein [58] that is ubiquitous in proliferating cells. The tandem bromodomains of Brd4 interact with acetylated tails of H3 and H4 histones [59], and Brd4 has been shown to be a mitotic bookmark that marks genes, which are expressed shortly after mitotic exit [60,61]. Brd4 decompacts chromatin and recruits transcriptional initiation and elongation factors to rapidly activate early G1 genes post-mitosis, as well as later in interphase [62]. Brd4 recruits the transcriptional elongation factor, p-TEFb, to promoters to enhance phosphorylation of the C-terminal tail (CTD) of RNA polymerase II promoters to stimulate transcription [63,64]. Brd4 further promotes transcription by directly phosphorylating the RNA polymerase II CTD [65]. This fundamental role of Brd4 in transcriptional regulation places it at the center of many diverse biological activities. 

**Figure 3:**
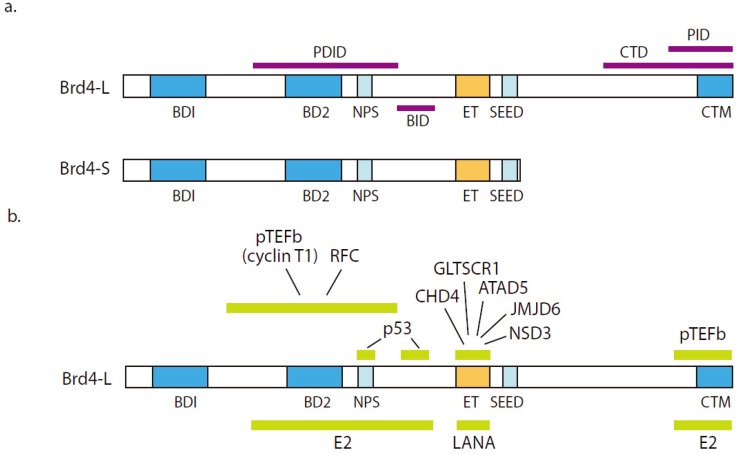
Structure and function of the Brd4 protein. (**a**) Domains of the Brd4 protein. The domains shown are BD1 (bromodomain I: residues 58–169); BD2 (bromodomain 2: residues 349–461); NPS (N-terminal cluster of phosphorylation sites: residues 484–503); BID (basic residue enriched interaction domain: residues 524–579); ET (extra-terminal domain: residues 600–678); PDID (phosphorylation dependent interaction domain: residues 287–530); SEED (Ser/Glu/Asp-rich region: residues 695–720); CTM (C-terminal motif: residues 1325–1362); CTD (dominant negative domain of Brd4: residues 1047–1362) and PID (pTEF binding region: residues 1209–1362) [73]. (**b**) Viral and cellular interacting partners of Brd4. See text for references.

The Brd4 gene encodes two proteins; the short form of Brd4 contains the two bromodomains and the extra-terminal (ET) region (important for many protein-protein interactions), while the longer form of Brd4 has an additional, long unique C-terminal region (see Figure 3). The structures of both bromodomains and the ET domains have been solved [66,67]. The bromodomains bind to specific acetylated lysines on H3 and H4, but BD2 (bromodomain 2) can also interact with acetylated residues in other proteins, such as cyclin T1 (the p-TEFb subunit) and the relA subunit of NFκB [67,68,69]. 

The extra-terminal domain consists of three alpha-helices, and it seems to be a site of protein-protein interaction [70]. It binds proteins involved in transcriptional regulation, such as NSD3 (also known as WHSC1L1), a histone methyl transferase, JMJD6, a histone demethylase, and CHD4, a component of the NuRD (nuclear remodeling and deacetylase) repressor complex. Thus, Brd4 has the potential to assemble multifaceted positive and negative regulatory complexes on promoters [71]. 

The function of the C-terminal region is not well-characterized, except for the last 100 amino acids, which is important for interacting with the papillomavirus E2 protein [72] and with p-TEFb [73] (see Figure 3). Thus, p-TEFb interacts with two independent regions of the Brd4 protein [68]. 

p53 has also recently been shown to be a binding partner of Brd4, and analysis of this interaction revealed a conformational switch in Brd4 structure and protein-protein interactions modulated by CK2 phosphorylation [74]. A region between the second bromodomain and the ET domain contains two interaction regions, BID (basic residue enriched interaction domain) and NPS (N-terminal cluster of phosphorylation sites), that modulate this switch. When unphosphorylated, the NPS region interacts with the PDID (phosphorylation dependent interaction domain) that encompasses bromodomain 2, thus preventing Brd4 from binding to acetylated histones (see Figure 3). p53 binds to the BID region when Brd4 is unphosphorylated. Upon phosphorylation of NPS, p53 is released from binding to BID, and both p53 and BID now associate with phosphorylated NPS, thus exposing the bromodomain 2 in PDID and activating the complex [74]. 

### 3.2. Association of Brd4 with Disease

The first association of Brd4 with human disease was the discovery that the aggressive NUT midline carcinoma (NMC) was due to a translocation (t(15;19)(q13;p13)), resulting in the fusion of Brd4 with NUT (nuclear protein in testes) [75]. The NUT protein recruits the histone acetyl transferase, p300, resulting in a feed-forward loop of histone acetylation and Brd4-NUT recruitment, which gives rise to hyperacetylated, but inactive, chromosomal foci [76]. Sequestration of Brd4 and p‑TEFb to these foci promotes proliferation of Brd4-NUT cells. This can be reversed and differentiation restored, by siRNA to Brd4, by histone deacetylase inhibitors that promote global acetylation and by BET protein-specific histone mimics [77,78,79]. 

Brd4 is also a modifier of breast cancer metastasis [80] and a promising target for several cancers, because of its fundamental role in transcriptional processes. Targeted inhibition of BET bromodomain binding is potentially therapeutic for glioblastoma, lung adenocarcinoma, ALL (acute lymphoblastic leukemia) and MLL (mixed-lineage leukemia) [81,82,83,84,85]. 

## 4. Brd4 and Papillomaviruses

Brd4 was discovered to be a major interactor of the papillomavirus E2 protein by proteomic analyses [20,72], yeast two hybrid screening [86]. It was also investigated as an E2 target, because the analogous tethering protein in KSHV (LANA) interacts with the BET family member, Brd2 [87,88,89]. The E2 protein had previously been shown to bind and tether viral genomes to host mitotic chromosomes [37,38,39,90], and the Brd4 protein colocalized completely with these chromatin bound speckles of E2 [72,87,89,91]. The E2 protein is a multifunctional protein involved in papillomavirus transcription, maintenance and partitioning of extrachromosomal viral genomes and initiation of viral DNA replication. Many subsequent studies have dissected the role of Brd4 in each of these processes. 

### 4.1. Interaction between Papillomavirus E2 and Brd4 Proteins

The Brd4 protein binds primarily to the transactivation domain of the E2 proteins (see Figure 2) [72]. The transactivation domain contacts a peptide at the extreme C-terminus of the Brd4 protein [91], and a C-terminal domain of Brd4 (CTD; residues 1047–1362) has proven useful as a dominant-negative inhibitor of the E2-Brd4 interaction [72]. The region of the E2 transactivation domain that makes contact with the Brd4 CTD is highly conserved; yet, there is a wide range of binding affinities between Brd4 and E2 proteins from different papillomaviruses [92]. The C-terminal DNA binding domain of E2 does not seem necessary for Brd4 binding, but the dimerization function of this domain greatly increases E2-Brd4 binding both *in vivo* and *in vitro*. There are hints that there may be additional contacts between E2 and Brd4: HPV11 E2 binds to the Brd4 CTD, but also to a region encompassing bromodomain 2 (BD2). The DNA binding domain of the KSHV LANA protein (a tethering protein analogous to E2 and with a DNA binding domain of similar structure) interacts with the ET domain of Brd4 [93,94]. It is likely that the interaction between Brd4 and the E2 protein will be multifactorial. 

### 4.2. Brd4 Modulates the Stability of the E2 Proteins

Several groups have noted that the E2 protein is stabilized by interaction with Brd4 [95,96] or with the Brd4 CTD [97]. This interaction prevents proteasomal degradation of E2 by the E3 ligase cullin‑3 [97] and may enhance many E2 functions, such as transcriptional regulation and stable tethering of genomes on host chromatin. 

### 4.3. The Role of Brd4 in Viral Transcription

The E2 protein can both activate or repress viral transcription, depending on whether it binds to sites that are distal or proximal to promoter elements [5,8,9,10]. Binding of E2 to promoter proximal sites represses the early viral promoter in the oncogenic alpha-PVs [11,12,13]. A number of mutational analyses identified residues in the transactivation domain of E2 that were important for E2‑mediated transcriptional activation or replication [98,99,100,101,102,103]. Prominent were two highly conserved residues (R37 and I73) that when mutated, abrogated E2-mediated transactivation, but not replication. Subsequently, it was shown that these residues were located on two adjacent alpha helices on the same face of the E2 transactivation domain [104], and later, these residues were shown to make direct contact with a C-terminal peptide (residues 1343–1362) of Brd4 [91]. In addition to binding to the Brd4 C-terminus, HPV11 E2 also interacts with Brd4 residues 280–580, which encompasses the BD2 bromodomain. 

It became clear that Brd4 was essential for the transcriptional activation function of E2. A dominant negative C-terminal peptide encompassing the Brd4 CTD interfered with the E2-Brd4 interaction and inhibited transactivation by many papillomavirus E2 proteins [92,105,106]. Initially, the role of Brd4 in transcriptional repression was controversial, but it has now been proven that Brd4 is also involved in E2-mediated transcriptional repression [18,19,20]. However, in some cases, the dominant negative Brd4 CTD interferes with E2-mediated transactivation, but not repression, implying that there are additional or alternative modes of interaction between the Brd4 and E2 proteins [18]. Another factor that contributes to repression of the HPV early promoter is the histone acetyl transferase complex, NuA4/TIP60 [18,107]. TIP60 preferentially acetylates K14 of histone H3 and K5, K8, K12 and K16 of histone H4 [108], which are all targets of the Brd4 bromodomains [66]. Notably, the HPV E6 protein destabilizes TIP60, thereby alleviating repression of its own promoter [107]. 

One of the key functions of Brd4 is to recruit p-TEFb to promoters to stimulate elongation of RNA polymerase II transcription. The C-terminal region of Brd4 interacts with both E2 and p-TEFb, suggesting that these complexes are mutually exclusive. Brd4 recruitment of P-TEFb to the early promoter is required for viral transcription, and E2 disrupts this interaction [109]. In this study (unlike [18]), the dominant negative BRD4 CTD was able to inhibit E2-mediated repression. 

Most HPV repression studies have analyzed viral transcription in cervical carcinoma-derived cell lines that harbor integrated HPV genomes. However, E2 may preferentially repress integrated genomes compared to episomally replicating genomes, and so, further studies are needed to define the role of Brd4 in HPV transcription [110].

### 4.4. The Role of Brd4 in Viral Genome Replication

Most studies have indicated that the E2-Brd4 interaction is important for transcriptional regulation and tethering of viral genomes to host chromatin. Mutated E2 proteins that are unable to bind Brd4 are able to efficiently support transient replication of an origin containing plasmid [87,98,99,105,106,111,112,113]. Ilves *et al.* demonstrated that the dominant negative Brd4 CTD could inhibit the replication of BPV1 genomes or origins in rodent cells, but not in human C-33A cells [113]. Furthermore, this inhibition was not dependent on the interaction of Brd4 with E2 (at least through the R37 and I73 residues) and was not specific for papillomavirus replication [113]. 

Wang *et al.* find that Brd4 is recruited to foci formed by HPV16 E1 and E2 in a replication origin‑dependent fashion in C-33A cells [114]. An E2 protein mutated in both Brd4 interacting residues (R37 and I73) is defective in replication, thus leading the authors to propose that Brd4 is required for replication. However, as previously found by others, E2 proteins with a single substitution in I73 are not defective in replication, despite an inability to bind to Brd4 [87,99,105,106,111,112,113]. Because downregulation of Brd4 has detrimental effects on cell growth and proliferation (making it difficult to interpret HPV replication experiments), Wang *et al.* demonstrated that Brd4 could stimulate HPV replication *in vitro* [114].

Somewhat similarly, we find that Brd4 is recruited to replication foci formed by the E1-E2 proteins in keratinocytes [49], in a process that is completely dependent on E1, E2 and Brd4. However, we find that Brd4 is displaced to the periphery of these foci in the presence of an actively replicating origin or genome, and Brd4 is no longer required for their formation [115]. Brd4 is also found in a satellite pattern around late replication foci that contain amplified genomes in differentiated keratinocytes [115], but it does not seem to be essential for viral DNA amplification [116]. The HPV replication foci induce a cellular DNA damage response and recruit repair proteins [46,47,48,49,50], and it is tempting to speculate that Brd4 is involved in these processes. The replication factor, RF/C, is found in Brd4 [117] and E2-Brd4 protein complexes [20,118]. Furthermore, the alternative RFC1 subunit, ATAD5, which is involved in the DNA damage response, interacts with the ET domain of Brd4 [71], suggesting that it might play a role in HPV replication. Clearly, more studies are required to elucidate the exact role of Brd4 in the papillomavirus replication process, but there are several hints that Brd4 might be involved in viral and cellular DNA replication and repair processes. 

### 4.5. The Role of Brd4 in Viral Genome Maintenance and Partitioning

**Figure 4:**
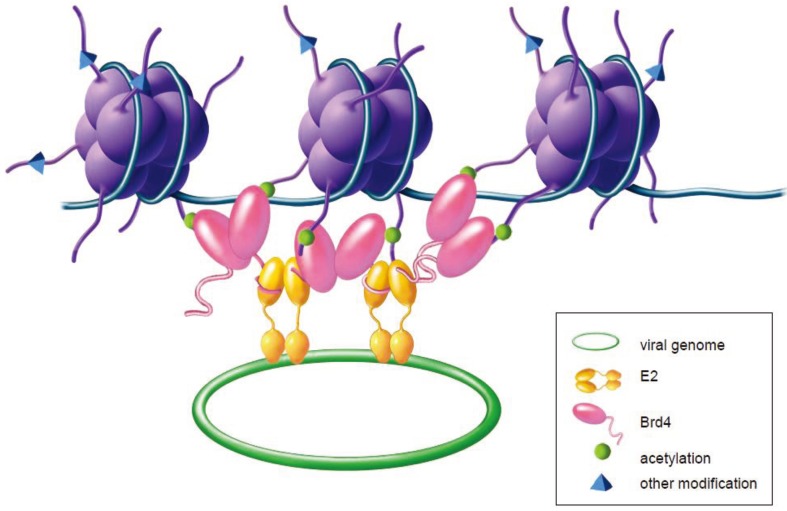
Model of E2-mediated tethering of the viral genome to host chromatin. The Brd4 protein interacts with acetylated lysine residues on histone tails protruding from the host nucleosomes (shown in purple). The papillomavirus E2 protein interacts with the C‑terminal region of Brd4 through the E2 transactivation domain. The DNA binding‑dimerization domain of E2 links the viral genome to the chromatin complex.

The E1 and E2 proteins support transient replication, but long-term persistence of viral-derived DNA requires additional E2 binding sites in *cis* to the replication origin [36]. The first clue to the role of E2 in viral DNA persistence was the observation that both viral DNA and the BPV1 E2 protein are localized in punctate foci on the host mitotic chromosomes [37]. This led to the model (as shown in Figure 4) that E2 associates with host chromosomes through the transactivation domain [39], while the DNA binding domain binds to E2 sites in the viral genomes and tethers them to the host chromosomes to promote retention and partitioning [119]. This tethering mechanism ensures that the low copy viral genome is retained in the nucleus and is partitioned to daughter cells.

At least for BPV1, the cellular target that mediates mitotic tethering is the Brd4 protein [72,87,89]. Brd4 is usually observed as a diffuse cloud (if at all) around the condensed mitotic chromosomes [59], but in the presence of E2, both proteins colocalize in punctate foci [89]. Brd4 has a high on-off rate and can be easily extracted from chromatin [59], but E2 dramatically stabilizes the interaction of Brd4 with chromatin forming a stable anchor in interphase and mitosis [89]. As described above, two highly conserved residues in the transactivation domain of E2 (R37 and I73) mediate the interaction with Brd4, and mutations in these residues abrogate the interaction of E2 with Brd4 and mitotic chromatin [87]. The DNA binding domain of E2 is not required for the interaction with Brd4, but it is required to link the viral genomes to chromatin [119]. The dimerization function of the E2 C-terminal domain greatly increases the affinity of Brd4 for chromatin both *in vivo* and *in vitro*, most likely by promoting the assembly of higher order E2-Brd4 complexes [120]. Expression of the dominant negative Brd4 CTD resulted in inhibition of E2 mitotic chromosome binding and loss of BPV1 genomes from BPV1 transformed cells [89,113,121]. Moreover, BPV1 E2-mediated plasmid maintenance could be reconstituted in *Saccharomyces cerevisiae* by exogenously expressed Brd4 [122].

Although the E2 proteins from all papillomaviruses interact with the C-terminal region of Brd4 (through the R37 and I73 residues in E2), there are differences in the strength of binding [92,118]. Furthermore, not all E2 proteins are as readily observed bound to mitotic chromosomes as BPV1 E2 [92,123]. A careful analysis of the E2 proteins from numerous different papillomaviruses showed that there were three different phenotypes of mitotic chromosome binding that segregated perfectly according to the phylogeny of papillomaviruses [123]. These groups were comprised of the alpha‑papillomaviruses, a large genus that encompasses mainly human viruses that infect the oral and genital mucosa; the beta and gamma papillomaviruses, another large group containing mainly human viruses that infect the cutaneous epithelium in an asymptomatic manner and a diverse group of viruses from the delta (BPV1), mu (HPV1), kappa (OcPV1 and SfPV1) and other genera. The E2 proteins from this latter group bind tightly to Brd4, stabilize its association with interphase chromatin and colocalize with Brd4 on the arms of mitotic chromosomes in punctate dots [92,123].

In contrast, the beta-PV E2 proteins bind strongly to pericentromeric regions of mitotic chromosomes that overlap the loci for the ribosomal RNA genes [123,124]. The determinants of the E2 protein required for this perichromosomal binding are quite different from those required for the E2‑Brd4 chromosomal foci. The primary requirements are the E2 DNA binding domain and a short peptide from the hinge region that facilitates interaction with chromatin [125]. Phosphorylation of this peptide by PKA (protein kinase A) stabilizes the E2 protein and promotes chromosomal binding [126] in a manner analogous to that of the KSHV LANA tethering protein [127]. However, the beta E2 proteins do have high affinity for Brd4, and if the chromosomal binding peptide in the hinge is mutated, E2-directed foci of Brd4 can be observed on mitotic chromosomes [128]. The significance of these two binding modes has yet to be determined, as beta-PV genomes do not readily replicate in cell culture.

For alpha-PVs, the mechanism of E2-mediated viral genome tethering and E2-Brd4 chromosomal binding is still elusive. Alpha-PV E2 proteins bind to Brd4 relatively weakly, they do not stabilize the association of Brd4 with host chromatin and cannot be easily detected on mitotic chromosomes, except in late telophase [123,129]. When cells are pre-extracted before fixation, the alpha-PV E2 proteins bind to the peri-centromeric regions of host chromosomes in a Brd4-independent manner similar to that of the beta-PVs [123]. Difficulties in detecting alpha-PV E2-Brd4 mitotic foci have led to the proposal of other targets, such as the mitotic spindle [130], a mitotic kinesin-like protein, MKlp2 [131], ChlR1 (an ATP-dependent DNA helicase important for sister chromatid cohesion) [132] and TopBP1 [129]. Furthermore, HPV31 genomes that encode a Brd4 binding defective E2 protein (an I73L mutation) can still maintain extrachromosomal viral genomes and undergo amplification in differentiated keratinocytes [54,105]. Recent findings indicate that Brd4 colocalizes with nuclear foci formed by the alpha-PV E1 and E2 proteins [114,115], and we find that the alpha-PV E1-E2 protein complex binds to the same regions of host chromatin as the stable HPV1 E2-Brd4 complex in C-33A cells [133].

Therefore, many questions remain, and it seems that the interaction of E2 and Brd4 with host chromatin is complex. The tethering mechanism is likely to be coupled with transcriptional and replication processes. Silla *et al.* have shown that simple attachment of genomes to chromatin is not sufficient [134]; chromatin attachment and transactivation functions must cooperate to ensure proper plasmid segregation. A genome-wide ChIP-on-chip analysis showed that BPV1 E2 and Brd4 were bound to transcriptionally active regions of chromatin, perhaps to ensure that the viral genome localized to transcriptionally active regions of the nucleus [135]. Brd4 is recruited to HPV replication centers containing alpha-PV proteins that do not tightly associate with Brd4 [114,115]. Thus, transcription, replication and genome partitioning are most likely intertwined processes. 

### 4.6. Association of Brd4 with Other Viruses

Papillomaviruses are not the only viruses that have discovered the versatility and usefulness of BET proteins [136]. The EBNA and LANA tethering proteins of the gamma herpes viruses, EBV and KSHV, interact with Brd2 and Brd4 for transcriptional regulation [88,93,137,138]. Polyoma viruses also recruit Brd4 to viral replication centers [139]. Brd4 represses HIV expression by competing with the HIV TAT transactivator for recruitment of p-TEFb to the HIV promoter [73]. Inhibition of Brd4 with BET inhibitors reactivates latent HIV with great therapeutic potential [140,141,142,143]. 

## 5. Conclusions

Viruses have always alerted us to the key players in cellular processes. Papillomaviruses, in particular, have small genomes with limited coding capacity and rely almost completely on using and manipulating cellular factors for viral processes. Brd4 is clearly a central player in HPV biology, and a complete understanding of its role in essential viral processes will provide deeper insight into its role in host biology.

## References

[1] Bouvard V., Baan R., Straif K., Grosse Y., Secretan B., El G.F., Benbrahim-Tallaa L., Guha N., Freeman C., Galichet L. (2009). A review of human carcinogens—Part B: Biological agents. Lancet Oncol..

[2] Walboomers J.M., Jacobs M.V., Manos M.M., Bosch F.X., Kummer J.A., Shah K.V., Snijders P.J., Peto J., Meijer C.J., Muñoz N. (1999). Human papillomavirus is a necessary cause of invasive cervical cancer worldwide. J. Pathol..

[3] Gillison M.L., Lowy D.R. (2004). A causal role for human papillomavirus in head and neck cancer. Lancet.

[4] Johansson C., Schwartz S. (2013). Regulation of human papillomavirus gene expression by splicing and polyadenylation. Nat. Rev. Microbiol..

[5] Spalholz B.A., Yang Y.C., Howley P.M. (1985). Transactivation of a bovine papilloma virus transcriptional regulatory element by the E2 gene product. Cell.

[6] McBride A.A., Byrne J.C., Howley P.M. (1989). E2 polypeptides encoded by bovine papillomavirus type 1 form dimers through the common carboxyl-terminal domain: Transactivation is mediated by the conserved amino-terminal domain. Proc. Natl. Acad. Sci. USA.

[7] Androphy E.J., Lowy D.R., Schiller J.T. (1987). Bovine papillomavirus E2 trans-activating gene product binds to specific sites in papillomavirus DNA. Nature.

[8] Cripe T.P., Haugen T.H., Turk J.P., Tabatabai F., Schmid P.G., Dürst M., Gissmann L., Roman A., Turek L.P. (1987). Transcriptional regulation of the human papillomavirus- 16 E6-E7 promoter by a keratinocyte-dependent enhancer, and by viral E2 trans-activator and repressor gene products: Implications for cervical carcinogenesis. EMBO J..

[9] Chin M.T., Hirochika R., Hirochika H., Broker T.R., Chow L.T. (1988). Regulation of human papillomavirus type 11 enhancer and E6 promoter by activating and repressing proteins from the E2 open reading frame: Functional and biochemical studies. J. Virol..

[10] Steger G., Corbach S. (1997). Dose-dependent regulation of the early promoter of human papillomavirus type 18 by the viral E2 protein. J. Virol..

[11] Thierry F., Yaniv M. (1987). The BPV1-E2 trans-acting protein can be either an activator or a repressor of the HPV18 regulatory region. EMBO J..

[12] Bernard B.A., Bailly C., Lenoir M.C., Darmon M., Thierry F., Yaniv M. (1989). The human papillomavirus type 18 (HPV18) E2 gene product is a repressor of the HPV18 regulatory region in human keratinocytes. J. Virol..

[13] Romanczuk H., Thierry F., Howley P.M. (1990). Mutational analysis of cis elements involved in E2 modulation of human papillomavirus type 16 P 97 and type 18 P 105 promoters. J. Virol..

[14] Tan S.H., Gloss B., Bernard H.U. (1992). During negative regulation of the human papillomavirus-16 E6 promoter, the viral E2 protein can displace Sp1 from a proximal promoter element. Nucleic Acids Res..

[15] Dong G., Broker T.R., Chow L.T. (1994). Human papillomavirus type 11 E2 proteins repress the homologous E6 promoter by interfering with the binding of host transcription factors to adjacent elements. J. Virol..

[16] Tan S.H., Leong L.E., Walker P.A., Bernard H.U. (1994). The human papillomavirus type 16 E2 transcription factor binds with low cooperativity to two flanking sites and represses the E6 promoter through displacement of Sp1 and TFIID. J. Virol..

[17] Nishimura A., Ono T., Ishimoto A., Dowhanick J.J., Frizzell M.A., Howley P.M., Sakai H. (2000). Mechanisms of human papillomavirus E2-mediated repression of viral oncogene expression and cervical cancer cell growth inhibition. J. Virol..

[18] Smith J.A., White E.A., Sowa M.E., Powell M.L., Ottinger M., Harper J.W., Howley P.M. (2010). Genome-wide siRNA screen identifies SMCX, EP400, and Brd4 as E2-dependent regulators of human papillomavirus oncogene expression. Proc. Natl. Acad. Sci. USA.

[19] Schweiger M.R., Ottinger M., You J., Howley P.M. (2007). Brd4 independent transcriptional repression function of the papillomavirus E2 proteins. J. Virol..

[20] Wu S.Y., Lee A.Y., Hou S.Y., Kemper J.K., Erdjument-Bromage H., Tempst P., Chiang C.M. (2006). Brd4 links chromatin targeting to HPV transcriptional silencing. Genes Dev..

[21] Thierry F., Howley P.M. (1991). Functional analysis of E2-mediated repression of the HPV18 P105 promoter. New Biol..

[22] Hwang E.S., Riese D.J.D., Settleman J., Nilson L.A., Honig J., Flynn S., DiMaio D. (1993). Inhibition of cervical carcinoma cell line proliferation by the introduction of a bovine papillomavirus regulatory gene. J. Virol..

[23] Dowhanick J.J., McBride A.A., Howley P.M. (1995). Suppression of cellular proliferation by the papillomavirus E2 protein. J. Virol..

[24] Desaintes C., Demeret C., Goyat S., Yaniv M., Thierry F. (1997). Expression of the papillomavirus E2 protein in HeLa cells leads to apoptosis. EMBO J..

[25] Stubenrauch F., Zobel T., Iftner T. (2001). The E8 domain confers a novel long-distance transcriptional repression activity on the E8E2C protein of high-risk human papillomavirus type 31. J. Virol..

[26] Chiang C.M., Broker T.R., Chow L.T. (1991). An E1M^E2C fusion protein encoded by human papillomavirus type 11 is a sequence-specific transcription repressor. J. Virol..

[27] Powell M.L., Smith J.A., Sowa M.E., Harper J.W., Iftner T., Stubenrauch F., Howley P.M. (2010). NCoR1 mediates papillomavirus E8;E2C transcriptional repression. J. Virol..

[28] Pett M., Coleman N. (2007). Integration of high-risk human papillomavirus: A key event in cervical carcinogenesis?. J. Pathol..

[29] Francis D.A., Schmid S.I., Howley P.M. (2000). Repression of the integrated papillomavirus E6/E7 promoter is required for growth suppression of cervical cancer cells. J. Virol..

[30] Jeon S., Allen-Hoffmann B.L., Lambert P.F. (1995). Integration of human papillomavirus type 16 into the human genome correlates with a selective growth advantage of cells. J. Virol..

[31] Mohr I.J., Clark R., Sun S., Androphy E.J., MacPherson P., Botchan M.R. (1990). Targeting the E1 replication protein to the papillomavirus origin of replication by complex formation with the E2 transactivator. Science.

[32] Ustav M., Stenlund A. (1991). Transient replication of BPV-1 requires two viral polypeptides encoded by the E1 and E2 open reading frames. EMBO J..

[33] Ustav M., Ustav E., Szymanski P., Stenlund A. (1991). Identification of the origin of replication of bovine papillomavirus and characterization of the viral origin recognition factor E1. EMBO J..

[34] McBride A.A. (2008). Replication and partitioning of papillomavirus genomes. Adv. Virus Res..

[35] Sanders C.M., Stenlund A. (1998). Recruitment and loading of the E1 initiator protein: An ATP-dependent process catalysed by a transcription factor. EMBO J..

[36] Piirsoo M., Ustav E., Mandel T., Stenlund A., Ustav M. (1996). Cis and trans requirements for stable episomal maintenance of the BPV-1 replicator. EMBO J..

[37] Skiadopoulos M.H., McBride A.A. (1998). Bovine papillomavirus type 1 genomes and the E2 transactivator protein are closely associated with mitotic chromatin. J. Virol..

[38] Ilves I., Kivi S., Ustav M. (1999). Long-term episomal maintenance of bovine papillomavirus type 1 plasmids is determined by attachment to host chromosomes, which is mediated by the viral E2 protein and its binding sites. J. Virol..

[39] Bastien N., McBride A.A. (2000). Interaction of the papillomavirus E2 protein with mitotic chromosomes. Virology.

[40] Kim K., Lambert P.F. (2002). E1 protein of bovine papillomavirus 1 is not required for the maintenance of viral plasmid DNA replication. Virology.

[41] Egawa N., Nakahara T., Ohno S., Narisawa-Saito M., Yugawa T., Fujita M., Yamato K., Natori Y., Kiyono T. (2012). The E1 protein of human papillomavirus type 16 is dispensable for maintenance replication of the viral genome. J. Virol..

[42] Xue Y., Bellanger S., Zhang W., Lim D., Low J., Lunny D., Thierry F. (2010). HPV16 E2 is an immediate early marker of viral infection, preceding E7 expression in precursor structures of cervical carcinoma. Canc. Res..

[43] Penrose K.J., McBride A.A. (2000). Proteasome-mediated degradation of the papillomavirus E2-TA protein is regulated by phosphorylation and can modulate viral genome copy number. J. Virol..

[44] Flores E.R., Lambert P.F. (1997). Evidence for a switch in the mode of human papillomavirus type 16 DNA replication during the viral life cycle. J. Virol..

[45] Sakakibara N., Chen D., McBride A.A. (2013). Papillomaviruses use Recombination Dependent Replication to Vegetatively Amplify their Genomes in Differentiated Cells. PLoS Pathog..

[46] Gillespie K.A., Mehta K.P., Laimins L.A., Moody C.A. (2012). Human papillomaviruses recruit cellular DNA repair and homologous recombination factors to viral replication centers. J. Virol.

[47] Moody C.A., Laimins L.A. (2009). Human papillomaviruses activate the ATM DNA damage pathway for viral genome amplification upon differentiation. PLoS Pathog..

[48] Fradet-Turcotte A., Bergeron-Labrecque F., Moody C.A., Lehoux M., Laimins L.A., Archambault J. (2011). Nuclear accumulation of the papillomavirus E1 helicase blocks S-phase progression and triggers an ATM-dependent DNA damage response. J. Virol..

[49] Sakakibara N., Mitra R., McBride A.A. (2011). The papillomavirus E1 helicase activates a cellular DNA damage response in viral replication foci. J. Virol..

[50] Reinson T., Toots M., Kadaja M., Pipitch R., Allik M., Ustav E., Ustav M. (2013). Engagement of the ATR-dependent DNA damage response at the human papillomavirus 18 replication centers during the initial amplification. J. Virol..

[51] Swindle C.S., Zou N., Van Tine B.A., Shaw G.M., Engler J.A., Chow L.T. (1999). Human papillomavirus DNA replication compartments in a transient DNA replication system. J. Virol..

[52] Van Doorslaer K., Tan Q., Xirasagar S., Bandaru S., Gopalan V., Mohamoud Y., Huyen Y., McBride A.A. (2013). The Papillomavirus Episteme: A central resource for papillomavirus sequence data and analysis. Nucleic Acids Res..

[53] Li R., Knight J., Bream G., Stenlund A., Botchan M. (1989). Specific recognition nucleotides and their DNA context determine the affinity of E2 protein for 17 binding sites in the BPV-1 genome. Genes Dev..

[54] Stubenrauch F., Lim H.B., Laimins L.A. (1998). Differential requirements for conserved E2 binding sites in the life cycle of oncogenic human papillomavirus type 31. J. Virol..

[55] Van Doorslaer K., Khan J., Chapman S., McBride A.A. (2013). Three E2 binding sites are sufficient for stable episomal maintenance of HPV18.

[56] Dey A., Ellenberg J., Farina A., Coleman A.E., Maruyama T., Sciortino S., Lippincott-Schwartz J., Ozato K. (2000). A bromodomain protein, MCAP, associates with mitotic chromosomes and affects G(2)-to-M transition. Mol. Cell Biol..

[57] Florence B., Faller D.V. (2001). You bet-cha: A novel family of transcriptional regulators. Front. Biosci..

[58] Houzelstein D., Bullock S.L., Lynch D.E., Grigorieva E.F., Wilson V.A., Beddington R.S. (2002). Growth and early postimplantation defects in mice deficient for the bromodomain-containing protein Brd4. Mol. Cell Biol..

[59] Dey A., Chitsaz F., Abbasi A., Misteli T., Ozato K. (2003). The double bromodomain protein Brd4 binds to acetylated chromatin during interphase and mitosis. Proc. Natl. Acad. Sci. USA.

[60] Dey A., Nishiyama A., Karpova T., McNally J., Ozato K. (2009). Brd4 marks select genes on mitotic chromatin and directs postmitotic transcription. Mol. Biol. Cell.

[61] Mochizuki K., Nishiyama A., Jang M.K., Dey A., Ghosh A., Tamura T., Natsume H., Yao H.J., Ozato K. (2008). The bromodomain protein Brd4 stimulates G(1) gene transcription and promotes progression to S phase. J. Biol. Chem..

[62] Zhao R., Nakamura T., Fu Y., Lazar Z., Spector D.L. (2011). Gene bookmarking accelerates the kinetics of post-mitotic transcriptional re-activation. Nat. Cell Biol..

[63] Jang M.K., Mochizuki K., Zhou M., Jeong H.S., Brady J.N., Ozato K. (2005). The bromodomain protein Brd4 is a positive regulatory component of P-TEFb and stimulates RNA polymerase II-dependent transcription. Mol. Cell.

[64] Yang Z., Yik J.H., Chen R., He N., Jang M.K., Ozato K., Zhou Q. (2005). Recruitment of P-TEFb for stimulation of transcriptional elongation by the bromodomain protein Brd4. Mol. Cell.

[65] Devaiah B.N., Singer D.S. (2013). Two faces of brd4: Mitotic bookmark and transcriptional lynchpin. Transcription.

[66] Filippakopoulos P., Picaud S., Mangos M., Keates T., Lambert J.P., Barsyte-Lovejoy D., Felletar I., Volkmer R., Muller S., Pawson T. (2012). Histone recognition and large-scale structural analysis of the human bromodomain family. Cell.

[67] Vollmuth F., Blankenfeldt W., Geyer M. (2009). Structures of the dual bromodomains of the P-TEFb-activating protein Brd4 at atomic resolution. J. Biol. Chem..

[68] Schröder S., Cho S., Zeng L., Zhang Q., Kaehlcke K., Mak L., Lau J., Bisgrove D., Schnölzer M., Verdin E. (2012). Two-pronged binding with bromodomain-containing protein 4 liberates positive transcription elongation factor b from inactive ribonucleoprotein complexes. J. Biol. Chem..

[69] Huang B., Yang X.D., Zhou M.M., Ozato K., Chen L.F. (2009). Brd4 coactivates transcriptional activation of NF-kappaB via specific binding to acetylated RelA. Mol. Cell Biol..

[70] Lin Y.J., Umehara T., Inoue M., Saito K., Kigawa T., Jang M.K., Ozato K., Yokoyama S., Padmanabhan B., Guntert P. (2008). Solution structure of the extraterminal domain of the bromodomain-containing protein BRD4. Protein Sci..

[71] Rahman S., Sowa M.E., Ottinger M., Smith J.A., Shi Y., Harper J.W., Howley P.M. (2011). The Brd4 extraterminal domain confers transcription activation independent of pTEFb by recruiting multiple proteins, including NSD3. Mol. Cell Biol..

[72] You J., Croyle J.L., Nishimura A., Ozato K., Howley P.M. (2004). Interaction of the bovine papillomavirus E2 protein with Brd4 tethers the viral DNA to host mitotic chromosomes. Cell.

[73] Bisgrove D.A., Mahmoudi T., Henklein P., Verdin E. (2007). Conserved P-TEFb-interacting domain of BRD4 inhibits HIV transcription. Proc. Natl. Acad. Sci. USA.

[74] Wu S.Y., Lee A.Y., Lai H.T., Zhang H., Chiang C.M. (2013). Phospho switch triggers brd4 chromatin binding and activator recruitment for gene-specific targeting. Mol. Cell.

[75] French C.A., Miyoshi I., Aster J.C., Kubonishi I., Kroll T.G., Dal Cin P., Vargas S.O., Perez-Atayde A.R., Fletcher J.A. (2001). BRD4 bromodomain gene rearrangement in aggressive carcinoma with translocation t(15;19). Am. J. Pathol..

[76] Reynoird N., Schwartz B.E., Delvecchio M., Sadoul K., Meyers D., Mukherjee C., Caron C., Kimura H., Rousseaux S., Cole P.A. (2010). Oncogenesis by sequestration of CBP/p300 in transcriptionally inactive hyperacetylated chromatin domains. EMBO J..

[77] Filippakopoulos P., Qi J., Picaud S., Shen Y., Smith W.B., Fedorov O., Morse E.M., Keates T., Hickman T.T., Felletar I. (2010). Selective inhibition of BET bromodomains. Nature.

[78] Schwartz B.E., Hofer M.D., Lemieux M.E., Bauer D.E., Cameron M.J., West N.H., Agoston E.S., Reynoird N., Khochbin S., Ince T.A. (2011). Differentiation of NUT midline carcinoma by epigenomic reprogramming. Canc. Res..

[79] Yan J., Diaz J., Jiao J., Wang R., You J. (2011). Perturbation of BRD4 protein function by BRD4-NUT protein abrogates cellular differentiation in NUT midline carcinoma. J. Biol. Chem..

[80] Crawford N.P.S., Alsarraj J., Lukes L., Walker R.C., Officewala J.S., Yang H.H., Lee M.P., Ozato K., Hunter K.W. (2008). Bromodomain 4 activation predicts breast cancer survival. Proc. Natl. Acad. Sci. USA.

[81] Lockwood W.W., Zejnullahu K., Bradner J.E., Varmus H. (2012). Sensitivity of human lung adenocarcinoma cell lines to targeted inhibition of BET epigenetic signaling proteins. Proc. Natl. Acad. Sci. USA.

[82] Ott C.J., Kopp N., Bird L., Paranal R.M., Qi J., Bowman T., Rodig S.J., Kung A.L., Bradner J.E., Weinstock D.M. (2012). BET bromodomain inhibition targets both c-Myc and IL7R in high-risk acute lymphoblastic leukemia. Blood.

[83] Dawson M.A., Prinjha R.K., Dittmann A., Giotopoulos G., Bantscheff M., Chan W.I., Robson S.C., Chung C.W., Hopf C., Savitski M.M. (2011). Inhibition of BET recruitment to chromatin as an effective treatment for MLL-fusion leukaemia. Nature.

[84] Delmore J.E., Issa G.C., Lemieux M.E., Rahl P.B., Shi J., Jacobs H.M., Kastritis E., Gilpatrick T., Paranal R.M., Qi J. (2011). BET bromodomain inhibition as a therapeutic strategy to target c-Myc. Cell.

[85] Mertz J.A., Conery A.R., Bryant B.M., Sandy P., Balasubramanian S., Mele D.A., Bergeron L., Sims R.J. (2011). Targeting MYC dependence in cancer by inhibiting BET bromodomains. Proc. Natl. Acad. Sci. USA.

[86] Olejnik-Schmidt A.K., Schmidt M.T., Kedzia W., Gozdzicka-Jozefiak A. (2008). Search for cellular partners of human papillomavirus type 16 E2 protein. Arch. Virol..

[87] Baxter M.K., McPhillips M.G., Ozato K., McBride A.A. (2005). The mitotic chromosome binding activity of the papillomavirus E2 protein correlates with interaction with the cellular chromosomal protein, Brd4. J. Virol..

[88] Platt G.M., Simpson G.R., Mittnacht S., Schulz T.F. (1999). Latent nuclear antigen of Kaposi's sarcoma-associated herpesvirus interacts with RING3, a homolog of the Drosophila female sterile homeotic (fsh) gene. J. Virol..

[89] McPhillips M.G., Ozato K., McBride A.A. (2005). Interaction of bovine papillomavirus E2 protein with Brd4 stabilizes its association with chromatin. J. Virol..

[90] Lehman C.W., Botchan M.R. (1998). Segregation of viral plasmids depends on tethering to chromosomes and is regulated by phosphorylation. Proc. Natl. Acad. Sci. USA.

[91] Abbate E.A., Voitenleitner C., Botchan M.R. (2006). Structure of the papillomavirus DNA-tethering complex E2:Brd4 and a peptide that ablates HPV chromosomal association. Mol. Cell.

[92] McPhillips M.G., Oliveira J.G., Spindler J.E., Mitra R., McBride A.A. (2006). Brd4 is required for e2-mediated transcriptional activation but not genome partitioning of all papillomaviruses. J. Virol..

[93] Ottinger M., Christalla T., Nathan K., Brinkmann M.M., Viejo-Borbolla A., Schulz T.F. (2006). The Kaposi’s Sarcoma-Associated Herpesvirus LANA-1 interacts with the short variant of BRD4 and releases cells from a BRD4- and BRD2/RING3-induced G1 cell cycle arrest. J. Virol..

[94] You J., Srinivasan V., Denis G.V., Harrington W.J., Ballestas M.E., Kaye K.M., Howley P.M. (2006). Kaposi’s sarcoma-associated herpesvirus latency-associated nuclear antigen interacts with bromodomain protein Brd4 on host mitotic chromosomes. J. Virol..

[95] Lee A.Y., Chiang C.M. (2009). Chromatin adaptor Brd4 modulates E2 transcription activity and protein stability. J. Biol. Chem..

[96] Gagnon D., Joubert S., Senechal H., Fradet-Turcotte A., Torre S., Archambault J. (2009). Proteasomal degradation of the papillomavirus E2 protein is inhibited by overexpression of bromodomain-containing protein 4. J. Virol..

[97] Zheng G., Schweiger M.R., Martinez-Noel G., Zheng L., Smith J.A., Harper J.W., Howley P.M. (2009). Brd4 regulation of papillomavirus protein E2 stability. J. Virol..

[98] Winokur P.L., McBride A.A. (1992). Separation of the transcriptional activation and replication functions of the bovine papillomavirus-1 E2 protein. EMBO J..

[99] Sakai H., Yasugi T., Benson J.D., Dowhanick J.J., Howley P.M. (1996). Targeted mutagenesis of the human papillomavirus type 16 E2 transactivation domain reveals separable transcriptional activation and DNA replication functions. J. Virol..

[100] Abroi A., Kurg R., Ustav M. (1996). Transcriptional and replicational activation functions in the BPV1 E2 protein are encoded by different structural determinants. J. Virol..

[101] Breiding D.E., Grossel M.J., Androphy E.J. (1996). Genetic analysis of the bovine papillomavirus E2 transcriptional activation domain. Virology.

[102] Ferguson M.F., Botchan M.R. (1996). Genetic analysis of the activation domain of bovine papillomavirus protein E2:its role in transcription and replication. J. Virol..

[103] Cooper C.S., Upmeyer S.N., Winokur P.L. (1998). Identification of single amino acids in the human papillomavirus 11 E2 protein critical for the transactivation or replication functions. Virology.

[104] Antson A.A., Burns J.E., Moroz O.V., Scott D.J., Sanders C.M., Bronstein I.B., Dodson G.G., Wilson K.S., Maitland N.J. (2000). Structure of the intact transactivation domain of the human papillomavirus E2 protein. Nature.

[105] Senechal H., Poirier G.G., Coulombe B., Laimins L.A., Archambault J. (2007). Amino acid substitutions that specifically impair the transcriptional activity of papillomavirus E2 affect binding to the long isoform of Brd4. Virology.

[106] Schweiger M.R., You J., Howley P.M. (2006). Bromodomain protein 4 mediates the papillomavirus e2 transcriptional activation function. J. Virol..

[107] Jha S., Vande P.S., Banerjee N.S., Dutta A.B., Chow L.T., Dutta A. (2010). Destabilization of TIP60 by human papillomavirus E6 results in attenuation of TIP60-dependent transcriptional regulation and apoptotic pathway. Mol. Cell.

[108] Kimura A., Horikoshi M. (1998). Tip60 acetylates six lysines of a specific class in core histones *in vitro*. Gene. Cell..

[109] Yan J., Li Q., Lievens S., Tavernier J., You J. (2010). Abrogation of the Brd4-positive transcription elongation factor B complex by papillomavirus E2 protein contributes to viral oncogene repression. J. Virol..

[110] Bechtold V., Beard P., Raj K. (2003). Human papillomavirus type 16 E2 protein has no effect on transcription from episomal viral DNA. J. Virol..

[111] Baxter M.K., McBride A.A. (2005). An acidic amphipathic helix in the Bovine Papillomavirus E2 protein is critical for DNA replication and interaction with the E1 protein. Virology.

[112] Brokaw J.L., Blanco M., McBride A.A. (1996). Amino acids critical for the functions of the bovine papillomavirus type 1 E2 transactivator. J. Virol..

[113] Ilves I., Maemets K., Silla T., Janikson K., Ustav M. (2006). Brd4 is involved in multiple processes of the bovine papillomavirus type 1 life cycle. J. Virol..

[114] Wang X., Helfer C.M., Pancholi N., Bradner J.E., You J. (2013). Recruitment of brd4 to the human papillomavirus type 16 DNA replication complex is essential for replication of viral DNA. J. Virol..

[115] Sakakibara N., Chen D., Jang M.K., McBride A.A. (2013). The Brd4 Chromatin Adaptor Protein is displaced from nuclear foci as the HPV E2 protein switches from Transcriptional to Replicational Modes.

[116] Stubenrauch F., Colbert A.M., Laimins L.A. (1998). Transactivation by the E2 protein of oncogenic human papillomavirus type 31 is not essential for early and late viral functions. J. Virol..

[117] Maruyama T., Farina A., Dey A., Cheong J., Bermudez V.P., Tamura T., Sciortino S., Shuman J., Hurwitz J., Ozato K. (2002). A Mammalian bromodomain protein, Brd4, interacts with replication factor C and inhibits progression to S phase. Mol. Cell Biol..

[118] Jang M.K., McBride A.A. (2013).

[119] Abroi A., Ilves I., Kivi S., Ustav M. (2004). Analysis of chromatin attachment and partitioning functions of bovine papillomavirus type 1 E2 protein. J. Virol..

[120] Cardenas-Mora J., Spindler J.E., Jang M.K., McBride A.A. (2008). Dimerization of the papillomavirus E2 protein is required for efficient mitotic chromosome association and Brd4 binding. J. Virol..

[121] You J., Schweiger M.R., Howley P.M. (2005). Inhibition of E2 binding to Brd4 enhances viral genome loss and phenotypic reversion of bovine papillomavirus-transformed cells. J. Virol..

[122] Brannon A.R., Maresca J.A., Boeke J.D., Basrai M.A., McBride A.A. (2005). Reconstitution of papillomavirus E2-mediated plasmid maintenance in Saccharomyces cerevisiae by the Brd4 bromodomain protein. Proc. Natl. Acad. Sci. USA.

[123] Oliveira J.G., Colf L.A., McBride A.A. (2006). Variations in the association of papillomavirus E2 proteins with mitotic chromosomes. Proc. Natl. Acad. Sci. USA.

[124] Poddar A., Reed S.C., McPhillips M.G., Spindler J.E., McBride A.A. (2009). The human papillomavirus type 8 E2 tethering protein targets the ribosomal DNA loci of host mitotic chromosomes. J. Virol..

[125] Sekhar V., Reed S.C., McBride A.A. (2010). Interaction of the betapapillomavirus E2 tethering protein with mitotic chromosomes. J. Virol..

[126] Sekhar V., McBride A.A. (2012). Phosphorylation regulates binding of the human papillomavirus type 8 E2 protein to host chromosomes. J. Virol..

[127] Woodard C., Shamay M., Liao G., Zhu J., Ng A.N., Li R., Newman R., Rho H.S., Hu J., Wan J. (2012). Phosphorylation of the chromatin binding domain of KSHV LANA. PLoS Pathog..

[128] McBride A.A., van Doorslaer K. (2013).

[129] Donaldson M.M., Boner W., Morgan I.M. (2007). TopBP1 regulates human papillomavirus type 16 E2 interaction with chromatin. J. Virol..

[130] Dao L.D., Duffy A., van Tine B.A., Wu S.Y., Chiang C.M., Broker T.R., Chow L.T. (2006). Dynamic localization of the human papillomavirus type 11 origin binding protein E2 through mitosis while in association with the spindle apparatus. J. Virol..

[131] Yu T., Peng Y.C., Androphy E.J. (2007). Mitotic kinesin-like protein 2 binds and colocalizes with papillomavirus E2 during mitosis. J. Virol..

[132] Parish J.L., Bean A.M., Park R.B., Androphy E.J. (2006). ChlR1 is required for loading papillomavirus E2 onto mitotic chromosomes and viral genome maintenance. Mol. Cell.

[133] Jang M.K., Sakakibara N., McBride A.A. (2013). Papillomavirus Genomes associate with the Cellular Protein Brd4 to replicate at Fragile Sites in the Host Genome.

[134] Silla T., Mannik A., Ustav M. (2010). Effective formation of the segregation-competent complex determines successful partitioning of the bovine papillomavirus genome during cell division. J. Virol..

[135] Jang M.K., Kwon D., McBride A.A. (2009). Papillomavirus E2 proteins and the host BRD4 protein associate with transcriptionally active cellular chromatin. J. Virol..

[136] Weidner-Glunde M., Ottinger M., Schulz T.F. (2010). WHAT do viruses BET on?. Front. Biosci..

[137] Lin A., Wang S., Nguyen T., Shire K., Frappier L. (2008). The EBNA1 protein of Epstein-Barr virus functionally interacts with Brd4. J. Virol..

[138] Viejo-Borbolla A., Kati E., Sheldon J.A., Nathan K., Mattsson K., Szekely L., Schulz T.F. (2003). A Domain in the C-terminal region of latency-associated nuclear antigen 1 of Kaposi’s sarcoma-associated Herpesvirus affects transcriptional activation and binding to nuclear heterochromatin. J. Virol..

[139] Wang X., Li J., Schowalter R.M., Jiao J., Buck C.B., You J. (2012). Bromodomain protein Brd4 plays a key role in Merkel cell polyomavirus DNA replication. PLoS Pathog..

[140] Li Z., Guo J., Wu Y., Zhou Q. (2013). The BET bromodomain inhibitor JQ1 activates HIV latency through antagonizing Brd4 inhibition of Tat-transactivation. Nucleic Acids Res..

[141] Boehm D., Calvanese V., Dar R.D., Xing S., Schroeder S., Martins L., Aull K., Li P.C., Planelles V., Bradner J.E. (2013). BET bromodomain-targeting compounds reactivate HIV from latency via a Tat-independent mechanism. Cell Cycle.

[142] Zhu J., Gaiha G.D., John S.P., Pertel T., Chin C.R., Gao G., Qu H., Walker B.D., Elledge S.J., Brass A.L. (2012). Reactivation of latent HIV-1 by inhibition of BRD4. Cell Rep..

[143] Banerjee C., Archin N., Michaels D., Belkina A.C., Denis G.V., Bradner J., Sebastiani P., Margolis D.M., Montano M. (2012). BET bromodomain inhibition as a novel strategy for reactivation of HIV-1. J. Leukoc. Biol..

